# Nanosized Drug Delivery Systems in Gastrointestinal Targeting: Interactions with Microbiota

**DOI:** 10.3390/ph9040062

**Published:** 2016-09-29

**Authors:** Michail Karavolos, Alina Holban

**Affiliations:** 1 Aberdeen, Scotland AB15 5LZ, UK; 2 Department of Microbiology and Immunology, Faculty of Biology, University of Bucharest, Bucharest 77206, Romania; alina_m_h@yahoo.com; 3 Department of Science and Engineering of Oxide Materials and Nanomaterials, Faculty of Applied Chemistry and Materials Science, Politehnica University of Bucharest, Bucharest 011061, Romania

**Keywords:** antimicrobial, nanotechnology, pathogen, infection, gastrointestinal delivery, microbiota

## Abstract

The new age of nanotechnology has signaled a stream of entrepreneurial possibilities in various areas, form industry to medicine. Drug delivery has benefited the most by introducing nanostructured systems in the transport and controlled release of therapeutic molecules at targeted sites associated with a particular disease. As many nanosized particles reach the gastrointestinal tract by various means, their interactions with the molecular components of this highly active niche are intensively investigated. The well-characterized antimicrobial activities of numerous nanoparticles are currently being considered as a reliable and efficient alternative to the eminent world crisis in antimicrobial drug discovery. The interactions of nanosystems present in the gastrointestinal route with host microbiota is unavoidable; hence, a major research initiative is needed to explore the mechanisms and effects of these nanomaterials on microbiota and the impact that microbiota may have in the outcome of therapies entailing drug delivery nanosystems through the gastrointestinal route. These coordinated studies will provide novel techniques to replace or act synergistically with current technologies and help develop new treatments for major diseases via the discovery of unique antimicrobial molecules.

## 1. Introduction

As new techniques in science and engineering are rapidly emerging, the possibilities of novel therapeutic and health-impacting approaches are also raised. Indeed, the continuously shortening availability of effective antibacterial agents has necessitated a shift in our focus to develop new technologies and molecules that will combat disease. The science of nanosized materials, nanotechnology, offers numerous improvements in most wide interest areas, such as medicine, industry, ecology, and engineering, to name the most relevant. As biomedical applications on nanotechnology are intensively investigated, researchers strive to elucidate the mechanisms underlying the most intimate interactions between newly-introduced nanostructured particles and live medically-important organisms [1]. 

For us humans, contact with nanoparticles is unavoidable in many areas of the modern life. Nanostructures are widely found in our natural environment, daily care products, foods, and numerous pharmaceutical products. The physical contact with nanoparticles may occur through any possible means, from skin penetration, inhalation, ingestion, or inoculation; these materials easily reaching the internal parts of our body [2].

The contact of nanoparticles with our gastrointestinal tract is encountered very often and it is considered to have the potential of inducing drastic changes to our cells, tissues, and organs. From ingested food, to the use of daily products that can be easily ingested, and also to the use of nanosized drug delivery systems for therapy, our gastrointestinal tract is prone to be “influenced” by these nanomaterials in widely undiscovered ways [3].

One broad interest biomedical application of nanotechnology is the therapy of infectious diseases. Indeed, numerous nanosystems have proved to provide efficient antimicrobial effects, being active not only against known susceptible microbes, but also against resistant pathogens and biofilms [4,5] This observation has led to the development of a newly-expanding research field aiming to investigate the interaction of nanoparticles with our commensal bacterial species, also called resident microbiota. Recent advances in genomics and metagenomics of microbiota, within or on the human body, has signaled a new era in our understanding of commensal populations inhabiting various human body niches. Now we are able to identify where and how disturbances in the microbiota, or dysbiosis as it is referred to, may lead to the development of disease. This area of research is in urgent need of attention since natural selection against antibiotics is proving to be too great a hurdle to overcome. It is crucial to identify novel dysbiostatic drugs that will help, on one hand, to prevent dysbiosis and, on the other hand, to reduce the incidents of natural selection that lead to the development of antibiotic resistance in pathogenic bacteria [6].

Although the interactions between nanoparticles that reach the gastrointestinal tract and microbiota are far from being understood, there is now clear experimental evidence that nanoparticles may have debilitating effects against human microbiota. These effects are a direct result of the preferential killing of microbiome components and, thus, the induction of dysbiosis. The mechanisms of action of nanomaterials against gut microbiota are not yet clearly understood. However, certain studies have demonstrated that nanoparticles have a distinct impact among different species of probiotics and resident microorganisms. This has led to the idea that nanoparticles in the gastrointestinal tract may also impact on the homeostasis of the human body and may change the balance between health and disease through modifying the composition and/or properties of microbiota. On the other hand, recent results helped to formulate the hypothesis that microbiota components may impact on the fate and effects of nanoparticles within the gastrointestinal tract, thus influencing the outcome of the therapy and disease. This has been observed, for example, in the case of nanostructured drug delivery systems used to target a particular disease through the gastrointestinal route [7].

## 2. Gut Microbiota, Health, and Disease

### 2.1. The Complexity of the Human Microbiome

The elucidation of the human microbiome has signaled a new age in our understanding of how microbes interact with themselves and the host in order to establish health or disease. The publication of the human microbiome has also highlighted the vastness and complexity of the microbial population in the human gut, skin, and vagina and has signaled a new era in microbial research [8,9,10,11,12].

It is intriguing that the gene pool of the average human healthy microbiota is approximately 150 times larger than the analogous human gene complement [10]. Indeed, recent data demonstrate that the microbes residing in the healthy human body collectively account to approximately 100 trillion cells, ten-fold the number of human cells, and may encode up to 100-fold more unique genes compared to the human genome [11]. The vast majority of the genes corresponded to bacterial hosts suggesting that the human microbiome niche comprises a total of 1000 to 1150 different bacterial species, of which about 160 are prevalent in any specific individual at any time during healthy life [10]. Crucially for human health, the gut microbes contribute significantly to how we are able to harvest energy from food, and it has been shown that changes in the healthy gut microbiome may be associated with bowel diseases or obesity [13,14]. During life, the human microbiome diversity changes, from highly diverse at the beginning of life to more similar phyla later in adulthood [12]. Interestingly, it has been noted that the diversity discovered so far in various microbiome projects may actually be under-represented since these studies have only included individuals within specific habitats and not free-living communities [15]. This last comment adds further credibility and urgency in recent trends to develop new techniques to identify, culture, and constructively interfere with the microbiome.

### 2.2. The Role of Microbiome Homeostasis: Dysbiosis and Disease

The homeostasis of the microbiome is maintained through a finely-controlled exchange of molecular information among the great number of microorganisms that form the specific population at any given niche. Indeed, the human microbiome is a signaling port that orchestrates many different environmental inputs, such as diet, with various genetic and immune signals to modulate the host’s metabolism, immunity and response to infection [16]. Deviations in the communication between the host microbiota and the innate immune system may, hence, contribute to complex diseases [16].

The connection between dysbiosis and disease has been well documented before [14,17,18]. The significance of microbiome balance is continuously being highlighted through old and new research. It is now accepted that disruption of microbiome normal function can, and usually does, lead to the development of disease. It is through either the introduction of pathogenic microbial agents or the elimination of normal commensal flora through the use of antibiotics that may result in the disruption of this delicate niche balance and lead to dysbiosis. Starting from an early stage in life, infant immune systems have been shown to respond to changes in microbiota composition to modulate their future response as adults in pathological conditions such as eczema, allergic and inflammatory disorders, including food hypersensitivity [19].

**Figure 1:**
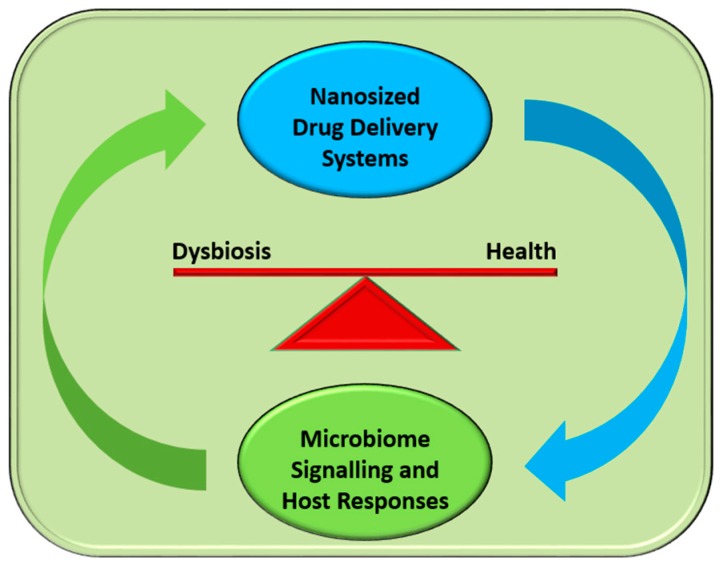
The intimate interactions between microbiome and nanomaterials. The complex biological signalling exchanges unfolding regularly during the encounters of drug-transporting nanoparticles and microbiota influence microbiome composition and, hence, the balance between health and disease (dysbiosis).

The improvements in cultivability of important microbiota candidates may lead to the advancement of novel therapies that will interfere with the microbiome in order to prevent dysbiosis (dysbiostatic drugs) and promote health [18,20]. Additionally, our ability to deliver nanosized particles effectively in the gut microbiome without adversely affecting its composition may provide valuable tools for the administration of novel drugs that will prevent dysbiosis and help to successfully treat diseases [21]. This capacity of various nanocarriers has been demonstrated in successfully managing the dysbiotic skin microbiome, in particular acne, through the administration of nanoparticles [22]. A brief diagram of possible interactions during this crucial encounter is represented schematically in Figure 1.

## 3. Gut Pathogens and Gastrointestinal Infections and Treatment

### 3.1. Most Common Infections

The importance of microbiome imbalance in the development of disease by various pathogens has been highlighted in a number of recent studies. For example, *Clostridium difficile* is an anaerobic, spore-forming, toxin-secreting pathogen with a long history of causing disease and is recognized to be the most common etiologic agent of antibiotic-associated diarrhea [23]. *C. difficile* infection is, at present, the most common cause of health care-associated infections in the US, accounting for approximately 12% of these incidents [24]. Changes in the composition of the gut microbial population structure and function through inappropriate antibiotic use is now known to increase host susceptibility to colonization by *C. difficile* and the development of disease [23,25].

In another example, infection with the important gut pathogen *Salmonella enterica* serovar Typhimurium (*S. Typhimurium*) was greatly enhanced upon perturbation of the microbiome in mice using a derivative of the short-chain fatty acid butyrate, sodium phenylbutyrate (PBA) [26]. It was shown that this reduction in the ability of *S. typhimurium* to cause disease in mice was through a PBA-mediated increase in the population of intestinal lactobacillales and segmented filamentous bacteria [26].

Another important pathogen, *Chlamydia trachomatis*, is a major cause of genital infections contributing to a major health problem worldwide [27,28]. It has been observed that certain women are more prone to developing repeated or chronic *C. trachomatis* infections. A recent study has been able to associate the increased prevalence of anaerobes in the female genital tract, which is normally populated by lactobacilli, with the development of bacterial vaginosis. Interestingly, this was demonstrated to involve a metabolic exchange regarding indole-producing anaerobes and the production of the amino acid tryptophan from *C. trachomatis*, which it then uses to aid its replication and overcome the host’s γ-interferon assault [27].

The list of possible microbiome-affected bacterial pathogens extends to further urinary tract infections, where a recent study has demonstrated the importance of the female urinary microbiota in the development of urinary tract infections [29]. It is suggested that previously unnoticed microbes and niche uniqueness may indeed be associated with urinary tract infection. In any specific case the function of individual microbiota may range from beneficial to pathogenic, and may differ on the basis of an individual’s female urinary microbiota uniqueness [29].

### 3.2. Dysbiosis: Treating the Imbalance

The gut can become a reservoir of potentially antibiotic-resistant life-threatening pathogens due to the increasing pressure of antibiotic treatments. The inability to treat the disease with antibiotics has led to the development of alternative treatment methods including, fecal microbiota transplantation (FMT). This practice is increasingly being adapted into many hospitals worldwide as a “last resort” treatment [30,31]. The gut microbiota has long been recognized as key players in the establishment of human health and disease. Many centuries ago, in 400 B.C., Hippocrates stated that, “…death sits in the bowels…” and “…bad digestion is the root of all evil.”[32].

FMT involves controlled administration of healthy donor fecal microbiota resulting in the establishment of a new colonization status and, hence, restoration of healthy gut microbial niche composition and function [30]. The mechanism behind FMT resides in the ability of the healthy donor microbiota to directly compete with the pathogen, in this case *C. difficile* and, hence, act in cohort with the immune system to clear the disease [30,32]. Furthermore, the metabolic action of the commensal microbiota delivered by FMT may contribute to the restoration of secondary bile acid metabolism in the colon resulting in a wider stimulation of the mucosal immune system and repair of the gut barrier [30,31].

A constantly increasing number of studies have now produced results to support the hypothesis that FMT poses a reliable method of interfering with a number of pathogens causing disease, including β-lactamase-producing and carbapenemase-producing Enterobacteriaceae, vancomycin-resistant *Enterococci*, as well as methicillin-resistant *Staphylococcus aureus* [33]. Current data are very promising for long-term clinical use of FMT since there were no adverse events even in immunocompromised patients, and additional trials are currently in progress to further explore the potential of FMT as an effective dysbiostatic treatment [33]. 

Reinstatement of a healthy microbial community structure through the clinical use of FMT practices may represent a reliable alternative to antibiotics when it comes to protecting against gut pathogen infections in general. FMT not only represents a sound alternative to pathogen control but could also be considered for the clinical treatment of chronic diseases, such as obesity, eczema, and allergic and inflammatory disorders that are commonly associated with certain conditions of microbiome dysbiosis.

## 4. Antimicrobial Nanosystems for Gastrointestinal Drug Delivery–Impact on Microbiota

A high number of drugs, from insulin to cancer chemotherapy, can be delivered only via injections. Although this delivery proved to be the only efficient choice for some drugs, it comes often with numerous disadvantages, including increased difficulty for patients, increased price, administration procedure and location (requiring trained medical personnel and, many times, having to be administrated in a healthcare facility). In infection control, antibiotics may be administered both by injection and as a pill, the choice being influenced by the type, location, and severity of the infection, but also by the particularities of the drug. The convenience of oral therapy continues to propel voracious interests in oral drug delivery and new approaches to improve the bioavailability of substances, including narrow absorption window drugs [34]. Oral administration of antimicrobial drugs may induce various side effects and it has the major disadvantage of uncontrollable targeting, destroying both pathogenic and non-pathogenic microbiota. Nanotechnology offers potentially unlimited perspectives in oral drug therapy, making possible rigorous targeting and controlled release, stabilization of the antimicrobial agent [35], improvements in the absorption and availability of drugs, as well as positively influencing gastroretention [36]. 

### 4.1. Types

Numerous approaches may be utilized to improve oral administration of antimicrobial drugs; amongst the most investigated we will discuss the following: nanoparticles, lipid drug delivery systems, polymeric micelles, nano-drug delivery systems, micro-drug delivery systems, hydrogels, emulsions, solid dispersions, and gastroretentive drug delivery systems [34].

Although numerous nanosystems proved their antimicrobial effect, a specific targeting element for particular bacteria has not yet been developed. However, recent studies demonstrate that antibiotic-based nanoformulations seem to be the most efficient in targeting bacteria. For example, vancomycin represents one of the preferred ligands for bacteria-targeting nanosystems because of the high affinity of the antibiotic for the bacteria cellular wall. Still, antibiotic targeting approaches have major limitations mainly because they are not efficient for resistant bacteria, having poor affinity to the reprogrammed cell wall structure [37].

#### 4.1.1. Metal Nanoparticles

Various types of organic and inorganic nanoparticles have enhanced antimicrobial activity, being active on most investigated pathogenic bacterial and fungal species, but also on gut-related viruses. Their advantages in antimicrobial therapy are multiple; from high specificity, increased efficiency in terms of time and therapeutic effect, targeted activity, and low cytotoxicity for the host [38].

Silver nanoparticles (AgNPs) have been used as efficient antimicrobials in a number of applications, including internal infections, topical wound dressings, and coatings for consumer products and biomedical devices. Silver salts and colloidal silver suspensions were commonly used to combat infection prior to the development of modern antibiotics. Ingestion is a relevant route of exposure for AgNPs, whether occurring unintentionally via Ag dissolution from consumer products, or intentionally from dietary supplements. Due to their proved antimicrobial efficiency, AgNP have even been proposed as substitutes for antibiotics in animal feeds, but also in some human gut infections. This approach is preferred, because, unlike ingested antibiotics, AgNPs have no or very limited effect on the indigenous microbiome. These nanoparticles may be engineered in various sizes, shapes, and may exhibit different biochemical properties. Moreover, AgNPs can be easily coated with antimicrobial agents and targeting molecules, which may increase their specificity and efficiency. In vivo tests performed in a murine model proved that gut microbial communities are not significantly modified after a 28 day treatment with AgNPs. The approach using culture-independent sequencing of 16S rRNA gene fragments obtained from mice with repeated oral dosing of well-characterized AgNPs of two different sizes (20 and 110 nm) and coatings (PVP = poly(vinylpyrrolidone) and Citrate), revealed that the membership, structure, or diversity of the murine gut microbiome is not affected, which was not the case upon administration of broad-spectrum antibiotics [21]. There is no proof that Ag nanoparticles are able to preferentially kill pathogenic bacteria but since some studies reported that Ag nanoparticles do not significantly alter the gut microbial communities, maybe, depending on the ingested dose, they could or could not significantly modulate the composition of microbiota, which is composed of numerous species. 

Gold nanoparticles (AuNPs) are increasingly utilized in medical applications as imaging and therapeutic agents, particularly as anti-inflammatories and drug delivery agents in various diseases, including infection. AuNP may be used parenterally, topically, and enterically, due to their properties. Oral administration of coloidal gold proved to reduce toxicity associated with injectable administration, but still maintain the efficiency [39]. Clinical efficiency, gastrointestinal absorption, and toxicity depend on the particles size and the synthesis route, therefore, in the last 10 years the progress of synthesis and characterization methods significantly improved the outcome of these nanoparticles [2]. Although AuNP exhibit interesting antimicrobial activities against pathogenic bacteria, their impact on human microbiota is still largely unknown. Gold nanoparticles can be biosynthesized by various microorganisms (bacteria and fungi) inhabiting various ecological niches, such as soil and water [40], but also by microorganisms belonging to human microbiota. Various skin microbiota species, such as *Staphylococcus saprophyticus*, *Micrococcus luteus*, *Staphylococcus homnis*, *Staphylococcus gallinaium*, and *Providencia sp.* were shown to produce metal nanoparticles, i.e., like gold, silver, platinum, palladium, and copper. Among these, only species of *Providencia* were able to produce AuNPs in all tested cultivation conditions. 

Titanium dioxide nanoparticles (TiO_2_NPs) are often ingested during various modern alimentary habits, since they are contained in relatively high amounts in dyes and artificial colouring agents. TiO_2_NP are frequently administered in injectable solutions for various diseases, especially in cancer therapy. They can be clinically administered by inhalation or oral delivery but studies reported increased cytotoxicity through these administration routes [41]. Antimicrobial efficiency of TiO_2_NP was recently demonstrated [42], although the impact of such nanoparticles on human or animal microbiota are far from being understood. However, TiO_2_NP, along with zinc oxide, were proposed to be highly efficient antimicrobial agents for oral hygiene; these nanoparticles inhibit biofilm formation and are efficient against oral pathogens causing periodontal diseases, thus, against oral microbiota species [43]. 

Cerium oxide nanoparticles (CeO_2_NPs) can be easily ingested by humans, since they are introduced into water resources through the disposal of coatings, pigments, and paints. Available data regarding their cytotoxicity after ingestion are poor, but recent studies demonstrated that they may impact on the viability of some gut microbiota species. Antimicrobial efficiency of these nanoparticles was proved against *Escherichia coli*, but also eukaryotic microorganisms [44]. CeO_2_NPs significantly increase microbial cell hydrophobicity in a human-like in vitro gut microbiota model. Moreover, these nanoparticles significantly modify the EPM (the electrophoretic mobility) parameter (an indicator of the relative surface charge) and change sugar and protein content of the extracellular polymeric substance (EPS). Furthermore, CeO_2_NPs caused cells to decrease significantly in size for the entire duration of the experiment; cells were significantly smaller during the CeO_2_ treatment after 4–5 days of exposure. Together with TiO_2_NPs, CeO_2_NPs were also detected in various cosmetic products, such as sunscreens, although their cytotoxicity is controversial and the impact on skin and skin microbiota is currently unknown [45].

Zinc oxide nanoparticles (ZnONPs) are often utilized in cosmetic and medical applications. Their extensive use in ointments, creams and sunscreens has led to abundant research publications regarding their cytotoxicity and antimicrobial effects. Although their impact on skin microbiota is far from being understood, the antimicrobial effects of ZnONPs are widely accepted by the scientific community. Their antimicrobial properties are size, concentration, and species-specific, being active also against gastrointestinal pathogens, such as *Salmonella enterica* [5]. ZnONPs also decrease hydrophobicity and cellular size in a gut microbiota model, also reducing the pH value in the human-like colon bioreactor [45]. However, cytotoxicity tests have shown that ZnONPs impair membrane integrity or eukaryotic cells and modify proliferation and viability of the cells, thus limiting their possible applications in the current gastrointestinal therapy. 

#### 4.1.2. Carbon-Based Nanomaterials

Carbon-based nanomaterials (multi- or single-walled carbon nanotubes (MWCNTs, SWCNTs)) have multiple biomedical applications, being applied mainly as drug delivery systems, since they demonstrate great loading versatility and have extraordinary mechanical, thermal, conductive, and electrical properties. Additionally, ultrashort carbon-based nanotubes (CNTs) are currently utilized in imaging applications and radiotherapy [46]. From the food industry, the implementation of nanostructured materials with nutraceutic properties recently emerged in the last 10 years, and numerous nanomaterials were approved for human consumption in various food formulations. CNTs represent the second most utilized nanosystems for food products destined for human consumption, after silver nanoparticles. Antimicrobial and antiparasitic properties are well investigated and widely accepted, and some CNT nanosystems being currently administered through an oral route for therapy. Along with their impact on pathogenic bacteria, CNTs administered through the oral route demonstrated an antimicrobial effect against gut bacteria [47]. Although some secondary effects were observed after prolonged oral administration or after inhalation (i.e., inflammation), the effect can be significantly reduced by manipulation of their length, surface properties, and nature of bioactive load [48]. Studies made on typical human gut pathogenic and non-pathogenic microbes proved that CNTs have distinct antimicrobial impact against gut microbiota, depending on the diameter, length, and surface modifications of the nanotubes, but also microbial species and, potentially, Gram character [47]. All types of investigated CNTs could inhibit the survival rates of *Lactobacillus acidophilus*, *Bifidobacterium adolescentis*, *Escherichia coli*, *Enterococcus faecalis*, and *Staphylococcus aureus*. Moreover, nanotubes directly interact with the cell walls of bacteria, disrupt membrane integration, induce a large amount of intracellular constituent DNA and RNA release, and reduce the bacterial membrane potential. It was demonstrated that the mechanism of the antibacterial activity was attributable to diameter-dependent piercing and length-dependent wrapping of CNTs causing the lysis of microbial cell walls and membranes. Additionally, it was suggested that the antibacterial efficiency of CNTs can be associated with the morphology of bacteria, and these nanosystems could have a broad antimicrobial spectrum [47].

#### 4.1.3. Polymeric Nanoparticles

Polymeric nanoparticles are composed mainly of biocompatible and biodegradable polymers and the most investigated polymeric nanosystems are designed for controlled drug release [49]. Along with their increased biocompatibility and biodegradability, polymeric nanoparticles display greater bioavailability, better encapsulation, controlled release, and less toxic properties. Additionally, nanoencapsulation of various drugs can be done easily by using polymeric nanosystems. The most investigated nanoparticles for drug delivery, including antimicrobial agents, with impacts on gastrointestinal infections are based on poly lactic-co-glycolic acid (PLGA), poly lactic acid (PLA), chitosan, gelatin, polycaprolactone, and poly-alkyl-cyanoacrylates [50]. 

In gastrointestinal drug delivery, nanoparticles are rapidly trapped in, and rapidly removed by, mucus, making controlled release difficult at this level. Although the rapid secretion and shedding of gastrointestinal tract mucus can significantly limit the effectiveness of nanostructured pharmacological systems, several types of polymeric nanoparticles were engineered to penetrate the mucus barrier and ensure a prolonged and efficient drug release [3]. Although this ability of recently-engineered polymeric nanoparticles are very useful for specific gastrointestinal targeting, this property is less applied in antimicrobial therapy, since the colonization of mucus with bacteria compared to the gastrointestinal lumen is very low. However, the fact that nanosystems penetrate the mucus barrier and ensure a prolonged effect may be important for their future antimicrobial effect in other downstream gastrointestinal tract areas, or even for specifically targeting pathogens which are able to colonize gastric mucus, such as *Helicobacter pylori*. 

Matrix-based polymeric nanosystems are the simplest, easiest to produce on an industrial scale, and the most cost-effective materials to ensure extended-release dosage forms of antimicrobial drugs. Usually, in such matrices the drug is combined and made into granules with excipients to improve the release rate of the drug, the matrix having different mechanisms toward the controlled action according to the used polymer. The most investigated matrix materials are swellable hydrophilic materials which release the drug after hydration of the polymer and gel formation on the surface of the polymer. 

A particular group of matrix materials is represented by biodegradable materials. Theseslowly release the drug while being eroded by body fluids. In the particular case of matrix materials that retain their shape during their passage through the gastrointestinal tract, the drug is slowly released from the matrix by diffusion. These nanosystems were proved to prolong the residence time of the drug at the target site and significantly extend the absorption time [51].

Hydrophilic matrix nanosystems could bring numerous advantages in the delivery and controlled release of antimicrobial drugs, especially antibiotics, to a gastrointestinal-specific target. Moreover, it is believed that they represent competitive candidates for severe gastrointestinal infections, able to overcome severe side effects, especially those related with microbiota disruption [52]. Antibiotics have common adverse effect, such as dose-dependent gastrointestinal stimulation occurring in the upper gastrointestinal tract. Bypassing the absorption site in the upper gastrointestinal tract brings significant advantages, especially in sustained-release preparations with larger dosage. Recent approaches utilize pH-responsive hydrogels composed of polymeric backbones with ionic pendant groups, since variations of pH specifically occur in the gastrointestinal tract and, thus, the release of the drug can be controlled. Synthetic polymers, such as poly(acrylamide), poly(acrylic acid), poly(methacrylic acid), poly(diethylaminoethyl methacrylate), and poly(dimethylaminoethyl methacrylate), and natural polymers, such as albumin and gelatin, were tested to produce efficient pH susceptible matrix nanosystems and recent results revealed that they improve the antimicrobial efficiency of the tested antibiotics (i.e., ciprofloxacin, aminoglycosides) by ensuring a targeted and controlled release [51,53]. 

Poly(lactic-co-glycolic acid) (PLGA) recently received FDA approval in the pharmaceutical industry for human applications, being a synthetic biodegradable and biocompatible polymer. PLGA has numerous biomedical applications, but gained increasing attention in the pharmaceutical industry as a drug carrier. Its use in antimicrobial therapy presents a great potential for treating severe infections, difficult-to-reach infectious diseases, and also those produced by resistant pathogens and biofilm-embedded microorganisms. PLGA nanoparticles were demonstrated to improve pharmacological and pharmacokinetic behavior of the loaded antimicrobial drugs, such as rifampicin [54], azithromycin, clarithromycin [55], and clindamycin [56]. However, such nanosystems also proved their efficiency in delivering and improving antimicrobial activity of some natural antimicrobial compounds and formulations, such as eugenol and trans-cinnamaldehyde. PLGA nanoparticles loaded with these natural compounds revealed an improved antimicrobial activity of the loaded compounds against food-borne pathogens, such as *Salmonella spp*. and *Listeria spp*. [57]. Recent studies suggest that PLGA-based functional nanosystems are highly efficient and versatile in antimicrobial therapy and could be utilized to treat infections occurring along the entire gastrointestinal tract, from endodontic infections [58] to severe gut infections, as well as device-associated infections which involve biofilm formation [59]. PLGA nanoparticles loaded with levofloxacin proved to be more successful against biofilm-associated infections produced by *Escherichia coli* and *Pseudomonas aeruginosa*, as compared with classical antibiotic therapy. These nanoparticles are believed to employ a biphasic extended drug release profile, comprising rapid initial antibiotic release to produce a high local concentration and a slower extended release to maintain a sufficiently high antibiotic concentration, and this behavior explains the enhanced anti-biofilm effect.

Another synthetic biodegradable and biocompatible polymer-poly(trimethylene carbonate) (PTMC) proved the constant release of antibiotics in a manner independent of the pH. This polymer is gradually degraded by the enzyme lipase and holds promise for antibiotic-modified release having biocatalytically-based and antimicrobial properties [60]. Studies revealed that gentamicin-loaded PTMC discs showed a controlled antibiotic release and the nanosystem proved antimicrobial efficiency both in planktonic microbial cultures and biofilms. The same research group demonstrated that the treatment with gentamicin loaded PTMC nanosystem inhibit biofilms formed by *Staphylococcus aureus* with about 80% and their inhibitory efficiency is maintained for more than 14 days [61].

To increase the loading abilities and overall versatility, PTMC is often mixed with other polymeric substances to produce efficient antimicrobial nanosystems. For example, a poly(lactic acid)/poly(trimethylene carbonate) (PLA/PTMC) matrix was utilized to design films incorporated with cinnamaldehyde and showed increased antimicrobial properties. This functional polymeric matrix proved to be efficient against bacterial species which present a risk of severe gut infections and, along with its drug delivery properties, proved a great potential for the design of improved food packaging materials [62].

Poly(lactic acid) (PLA) is a bioabsorbable, non-toxic, and biodegradable polymer with numerous biomedical applications, with a great potential in drug delivery. PLA-based nanosystems were tested in various anti-infectious approaches and this polymeric material proved its anti-parasitic and anti-microbial efficiency. PLA nanoparticles were loaded with halofantrine hydrochloride, a new antimalarial drug, and supported a prolonged activity and a lower host toxicity of the drug, while maintaining increased anti-malaria effects [63]. Since oral delivery of drug-loaded PLAbased nanosystems is often applied, recent studies have been made to test its impact on the gut microbiota. Results showed that PLA may efficiently deliver antibiotics and antimicrobial drugs and may ensure selective antimicrobial effects. Nisin, an antimicrobial peptide of bacterial origin, which was recently approved by the Food and Drug Administration (FDA) for use as a food preservative, was loaded into PLA nanoparticles to overcome its stability and solubility disadvantages. The resulting polydispersable nanoparticles revealed a prolonged and active release of nisin from the PLA nanosystem and an intense antimicrobial effect against *Lactobacillus delbrueckii* [64].

Naturally-derived materials possess the advantage of similarity to the extracellular matrix, very good biocompatibility, and easy surface modification, but their application could be limited by inadequate mechanical properties and processability. Natural polymers are often surface-modified or used with other materials to enhance their properties and suitability for targeting drug delivery systems efficient for severe gastrointestinal infections [65]. Chitosan is a widely investigated polymer for the gastrointestinal delivery of antibiotics possessing a wide antimicrobial spectrum and high killing rate against Gram-positive and Gram-negative bacteria, but lower toxicity toward mammalian cells. This copolymer of *N*-acetylglucosamine and glucosamine derived from chitin has been included in European pharmacopoeia and US pharmacopeia and is widely utilized for therapeutic approaches due to its biocompatibility, mucoadhesion, and permeation-enhancing properties [66]. Chitosan proved to be a superior polymer for antibiotic delivery, with microencapsulation approaches often being used for the design of gastrointestinal drug delivery systems. Studies demonstrated that chitosan microspheres may be optimized for higher acid resistance by being crosslinked with pentasodium tripolyphosphate during microencapsulation. Such crosslinked chitosan microspheres proved to be more stable in simulated gastric fluid, and also ensured a slower release of antibiotics, especially ampicillin [67]. 

The main difference between polymeric nanosystems and nanoparticles with respect to their antimicrobial effect is that, usually, nanoparticles are more likely to have intrinsic antimicrobial effects (especially because of their small size and physicochemical properties), while polymeric nanosystems are basically not antimicrobial, but they are efficient carriers and drug delivery devices for antimicrobial agents. 

### 4.2. Mechanisms and Behavior in the Gastrointestinal Tract

As the gastrointestinal tract represents a likely route of entry for many nanomaterials, directly through intentional ingestion or indirectly via nanoparticle dissolution from food containers, or by secondary ingestion of inhaled particles, their mechanisms of action, advantages, and disadvantages should be widely known for an appropriate therapy. Additionnally, along with cytotoxicity and biodistribution studies, other aspects relating to nanoparticle oral intake are currently under investigation. The impact of nanoparticles on the host microbiota in the gastrointestinal tract is believed to play a key role in the outcome of any given therapy. Nanoparticles were proved to exhibit preferential damage on the microbiota but, also, it is believed that microorganisms of normal microbiota could influence toxicity of nanoparticles and their interaction with the host cells on site [7]. 

Although oral administration of functional nanoparticles is not a common event, nanoparticles come in contact with human microbiota very often through ingestion of solid food, water, cosmetics, and personal care products. Nanoparticles are contained as active ingredients by numerous cosmetic and daily care products, and their interaction with human microbiota is unavoidable. Among these, Ag, SiO_2_, TiO_2_, and ZnO nanoparticles are most relevant for oral ingestion because they are added as ingredients to food and many are contained in healthcare products. Numerous aspects, such as physicochemical properties, particle size, surface area, particle number, aggregation/agglomeration state, charge, and surface coatings are all likely to influence the biological effects and action mechanisms of orally-administered nanoparticles [68]. Moreover, oxidant generation and rate of dissolution were proved to impact the absorption of nanoparticles at the gastrointestinal level; therefore, these mechanisms are considered when investigating gastrointestinal drug delivery nanoparticles. Although not fully demonstrated, researchers consider that the mechanisms of action and impact of nanoparticles on the components of the gastrointestinal tract can vary due to resident microbial species, strain, diet, housing conditions, time of dosing, circadian rhythm variations, and endogenous microbiota [2]. These elements could also impact on the efficiency of functional nanoparticles in the treatment of gastrointestinal diseases, including infections.

#### 4.2.1. Absorption

After reaching the gastrointestinal tract, nanoparticles can be translocated through the intestinal barrier in a multistep process that involves diffusion through the mucus layer, contact with enterocytes and/or M-cells, and uptake via cellular entry or paracellular transport. The most common mechanism for uptake of NP into intestinal epithelial cells appears to be endocytosis with various mechanisms, such as clathrin-mediated, caveolae-mediated, clathrin and caveolae-independent, and macropinocytosis [69]. The uptake mechanism seems to be influenced by the physicochemical properties of the nanoparticles, their aggregative behavior, and size [70]. 

Studies have demonstrated that non-lymphoid areas can also be involved in the absorption of nanoparticles, particularly with conjugation of nutrients or nutrient-like compounds, which may increase their uptake. However, the major uptake via M-cells and the lymphoid system has not been addressed and also these combinations may employ side effects such as enhanced activation of pro-inflammatory pathways [71]. 

After absorption, ingested nanoparticles may have distinct biodistribution, mainly depending on their size, while smaller particles enter the bloodstream and accumulate in the liver and spleen, the larger nanoparticles remain in the submucosa of the gastrointestinal tract, or are not absorbed at all [2,70]. 

#### 4.2.2. Dissolution

The stability, dissolution, and release of potentially toxic ions are dependent on the pH and composition of the fluid in the gastrointestinal tract, but also on the duration of exposure. Major pH changes along the oro-gastrointestinal tract significantly influence the dissolution and subsequent mechanisms of ingested nanoparticles. At low pH the dissolution of nanoparticles is increased and enzymes in the digestive fluids can induce rapid denudation of particles. The influence of dissolution seem to vary among nanoparticles, according with their size, being more pronounced for smaller particles as they agglomerated to a higher degree [72]. Additionally, particle coating may play a key role in the dissolution and also in the release of active compounds. Acidic pH induces a faster dissolution of highly acidic soluble nanoparticles, such as ZnO, while nanostructures with lower solubility in acid solutions, such as SiO_2_, agglomerate in the gastric fluid and are more difficult to be subjected to dissolution [73]. Variation in the pH within the gastrointestinal compartments also alter the surface chemistry of nanoparticles, particularly in those where zeta potential is highly dependent on pH (e.g., chitosan) [74].

Understanding parameters of dissolution in gastrointestinal fluids may help predict uptake and blood concentrations of the bioactive compounds contained in the nanostructured drug delivery system. However, being a very complex and diverse environment, the mechanism of action of functional nanoparticles in the gastrointestinal tract is difficult to accurately predict and their physiological and chemical behavior in the gut is especially believed to play a very important role in the outcome of the disease treatment.

## 5. Conclusions and Perspectives

The exponential growth in microbiome research and the necessity to develop novel antimicrobial drugs that will prevent dysbiosis and, hence, disease has led to a number of novel entrepreneurial avenues with great potential. This review highlighted the current developments in nanotechnology in biomedicine and its relations to microbiota. Nanostructured materials bring a new perspective to numerous fields from industry to medicine, but their impact on human health is far from being understood. It is clear that extensive research has to be conducted in order to elucidate the relevant mechanisms and impacts of nanoparticles within the gastrointestinal route, on the gastrointestinal tract components, and especially their interaction with microbiota. Interestingly, recent studies bring fascinating new evidence revealing the possibility of clear and bi-oriented interactions between nanoparticles and members of the human microbiota. One clear conclusion is that any nanostructure that reaches or transit the gastrointestinal tract has the potential to interfere with microbiota and lead to dysbiosis, thus impacting on the health of the host and also on the outcome of the applied nanotherapy. This observation needs coordinated and highly focused attention since the scientific clarification of these pathways may provide new avenues for the discovery of novel treatments for disease which require gastrointestinal therapy.
